# On the Relation between the Gas-Permeability and the Pore Characteristics of Furan Sand

**DOI:** 10.3390/ma14143803

**Published:** 2021-07-07

**Authors:** Dinesh Sundaram, József Tamás Svidró, Judit Svidró, Attila Diószegi

**Affiliations:** Department of Materials and Manufacturing, Jönköping University School of Engineering, Gjuterigatan 5, Box 1026, SE-55111 Jönköping, Sweden; jozsef.svidro@ju.se (J.T.S.); judit.svidro@ju.se (J.S.); attila.dioszegi@ju.se (A.D.)

**Keywords:** Darcy’s law, furan sand, grain size, porosimetry, porosity, permeability, silica sand

## Abstract

Furan sand is one of the most commonly used chemically bonded molding materials in foundries across the world. It consists of a furfuryl alcohol-based resin and an acid-based liquid catalyst. When the molding material comes in contact with the molten metal, it undergoes a thermal shock accompanied by a certain release of volatile gases. In order to evacuate these gases, molds and cores should have optimal gas permeability values and proper venting by design. If the volatile compounds are not appropriately evacuated, they are prone to enter the melt before the first layer of solidified metal is formed which can lead to the formation of gas-related casting defects. Standard gas permeability measurements are commercially available tools used in the industry to compare and to quality control different sands, however, they only provide reference numbers without actual units. Permeability in a standard unit, m^2^, provides uniformity and helps the comparison of results from difference sources. In this paper, a new method using Darcy’s law (prevalent in earth sciences), was adapted to measure the gas-permeability of furan samples made of silica sand with various grain size distributions. The effect of grain size distribution on the gas-permeability of furan sand samples was studied. Gas-permeability values in m^2^ were then correlated with mercury-porosity measurement results to bring new light on the relation between pore size, pore volume and the permeability of molding materials.

## 1. Introduction

Sand casting is one of the earliest processes for producing castings. It is versatile, cheap, and allows high volume manufacturing of cast metal components. Molding mixtures, or often called sand systems, consist of granular refractory materials, resins or binders, and additives. Molding mixtures need to meet specific requirements in order to manufacture castings of good quality. Out of those, the requirement of good gas permeability is essential [1]. Due to the imminent thermal shock after pouring, gases and other volatiles try to find their way out of the mold cavity [2,3]. Depending on the gas-permeability of the mixture, gases will either escape the mold through the core prints or get trapped in the solidifying metal. Chemically bonded sands are used to produce cores primarily. Out of the many types, furan sand is the most prevalent no-bake mixture that is used in the foundry industry. It commonly consists of furfuryl alcohol-based resin and acid-based catalyst that accelerates the exothermic polymerization process of curing [1].

The standard test method for gas-permeability approved by the American Foundry Society results in a dimensionless number called permeability number [4]. The test procedure applies a modified version of Darcy’s law, where the time *t* a sample takes to let a certain volume of air pass through is measured. This permeability number can be used as a comparison tool to determine the differences in permeability between samples. The gas-permeability can be measured in standard units (m^2^) if the molding material is considered as a porous medium with a certain amount of porosity in its structure. Darcy’s law can then be applied to find the permeability coefficient, *K*, of the sample [5]. Darcy’s law is given in Equation (1).

(1) $$Q=\frac{K\;A\;dP}{\mu \;L}$$

The description of the symbols could be found in the Nomenclature section. The velocity of the fluid passing through the porous medium, Darcy velocity, is essential to model fluid flow mechanics in a porous medium. In the metal casting context, to model the mass and heat transport of the gases evolved during the casting process, it is imperative to measure the gas-permeability in standard units. In foundry technology, researchers have studied gas-permeability using different non-standard techniques, using Darcy’s law. Winardi et al. [6] measured the volumetric flow rate of different samples using a modified version of Darcy’s law. Wisniewski et al. [7] investigated the type of resin and its effect on the porosity and gas-permeability of ceramic molds. The work involved using a custom-made system for measuring gas-permeability using Darcy’s law in m^2^. Kumar et al. [8] studied the gas-permeability of ceramic molds by passing compressed air through the samples and recorded the pressure drop. When permeability is one of the many aspects measured, researchers have used the simple permmeter [8,9]. In this work, permeability is calculated using a custom-made setup, where air is passed through a standard cylindrical sample, and the flow properties such as the pressure drop and velocity are measured [10].

## 2. Background

### 2.1. Influence of Grain Size on the Permeability of Chemically Bonded Mixtures

Molds and cores have complex pore geometry due to the silica sand grains of multiple sizes. Besides their size, the shape of grains has a direct impact not only on the total porosity but also on the gas-permeability of the produced cores [3]. Sieve analysis of sand provides data about the different fractions of grain sizes in a refractory aggregate. From the data obtained from sieve analysis, it is possible to calculate the average grain size of the sand. The American Foundry Society has provided a standard test procedure for measuring the average grain size from the grain size distribution of a granular material [4]. In foundry technology, several authors have aimed to study the grain size distribution and its effect on permeability. Madi et al. [11] determined the grain size distributions of two commercially available silica sand. They studied the effect of varying resin quality on the evolution of gases from the sand cores at different casting temperatures. They observed that the gas pressure difference for medium sized grain samples were lower, while the fine sized grain samples showed a higher gas pressure. They also reported rapid increase in gas pressure for no-bake cores. Madi et al. [12] also studied the effect of varying grain sizes of the sand used to produce foundry cores on the pressure build-up in the cores. The authors showed how the peak pressure varies for samples with varying grain size distributions. Sahoo et al. [13] investigated the foundry properties of locally available sand. In their work, they emphasized the importance of the grain fineness number (GFN) as a critical foundry sand property and its effect on the permeability of prepared cores. Some authors have predicted the permeability with a certain level of accuracy using the diameter of the grains as they are of a defined size batch and as they have a certain grain size distribution [14]. The authors presented formulas for predicting permeability for different cases (binary or mixed sand).

The pore structure of molds and cores containing different sizes of sand grains is complex and comes with a pore size distribution. The pore size distribution of a porous material is of great interest to study the mass and heat transfer of the material and is applicable to model the flow characteristics of the material. Gas-permeability is one of the significant flow characteristics for foundry sand mixtures as it influences the potential defect formation in the castings. The porosity of a material is defined as the fraction of the total volume of the medium that is occupied by void space [15]. The pore size distribution is primarily dependent on the size of the grains that form the porous material. However, in the case of foundry cores and molds, it also depends on the compaction methods and forces. Mitra et al. [16] studied the mass transport properties of 3d-printed sand cores using X-ray micro-computed tomography and correlated the permeability results using a non-destructive method. They measured the permeability in Darcy units and obtained permeability numbers of the range 4.43 × 10^−11^–9.18 × 10^−11^ for samples with porosity ranging from 49–53%. Samples with varying binder content and varying average grain sizes were studied and reported in their work. The main parameters studied were the pore structure characteristics and permeability. Research studying the correlation between permeability and pore size distribution has been carried out primarily in the field of earth sciences. However, in foundry technology, very little light has been thrown in measuring the pore structure characteristics and its effect on permeability. Marshal et al. [17] studied the relationship between pore size distribution and the gas-permeability of different sand types. The relationship they established is given in Equation (2).

(2) $$K=\varepsilon^{2}n^{-2}\left[r_{1}^{2}+3r_{2}^{2}+5r_{3}^{2}+\cdots +\left(2n-1\right)r_{n}^{2}\right]/8$$

The description of the symbols could be found in the Nomenclature section. Ettemeyer et al. [18] published their work on creating a model for predicting the physical properties of sand cores by using X-ray micro-computed tomography technique and have compared the results of permeability, strength, and thermal conductivity with the simulations. In their study, they have used a test rig set up to measure the permeability of three different sand samples. They present the results of permeability based on the outlet velocity of the measured samples and compare it with the simulated results of permeability. Vaskova et al. [19] studied the effect of combining coarse sand with fine sand and reported a decrease in permeability with an increasing percentage of fines content. Their results show that after a certain amount of increased fines, the permeability did not reduce much for the sand mixtures. However, permeability was measured for loose sand with varying grain size distributions using a permmeter and according to standard AFS procedure, which resulted in dimensionless numbers. Secondly, the effect of variation in the grain size distribution on the pore structure is not studied. Hence the need for quantifiable data (with units) on pore structure and permeability becomes a necessity.

### 2.2. The Role of Pore Characteristics on the Permeability

The works cited thus far highlight the importance of grains size distribution of foundry cores and molds and its effect on the permeability. It is also important to study the pore characteristics of foundry cores to understand the flow behavior it exhibits. There are several methods to measure the pore structure of materials, including direct methods using the apparent and true densities [20], non-wetting liquid intrusion techniques, gas adsorption techniques, and microscopy/tomography techniques [21]. In this work, the mercury porosimetry technique was used to characterize the pore network in the furan samples. The technique is governed by the Washburn equation, which relates the pore radius to the external pressure applied to the sample [22].

(3) $$P=\frac{-2\gamma \cos\theta }{r}$$

The description of the symbols could be found in the Nomenclature section. Mercury intrusion porosimetry provides the true density, bulk density, porosity, and pore size distribution of the material studied. In this study, these properties were studied for furan sand mixtures. The use of mercury porosimetry on the samples was expected to establish the relationship between the variation in the grain size, porosity, and permeability of chemically bonded sand mixtures. It is worthy of note that the Washburn equation and, therefore, the mercury porosimetry technique assumes that the geometry of the pore is a cylinder. According to the size of the pores, the international union of pure and applied chemistry (IUPAC) has classified pores into three types [23]. Pores below 2 nm are called micro-pores. Pores of 2–50 nm are classified as mesopores, and pores larger than 50 nm are categorized as macro-pores. Fang et al. [24] used a relationship derived for the evaluation of absolute permeability of coals. The relationship is based on percolation theories developed in the geothermal fields using mercury intrusion porosimetry (MIP) data. Similarly, Dong et al. [25] estimated the air permeability of artificial sandstones that are prepared from quartz sand bonded by epoxy resin based on correlations found by Pittman [26].

The authors deduced the permeability of coal samples using the MIP data for different ranks of coal. Although MIP has been used extensively over the years for the characterization of pore networks in porous bodies, some authors [16] have been skeptical about the usage of this method on foundry sands. They mention that foundry sand is an unconsolidated material, and therefore they cite the potential danger of destructing the integrity of the sample and causing void collapse. However, authors such Martinez et al. have used the technique and published their results. Since the samples used in this study were chemically bonded sand, they could be deemed as consolidated materials to a certain extent. Martinez et al. [27] studied furan bound 3D-printed sand samples to analyze the effect of binder content on several properties of the samples such as density, dimensional accuracy, mechanical strength, and pore network characterization. They concluded that they did not find any significant differences in pore structure morphology due to varying binder concentrations. However, they note the difference in bulk permeability of the samples with varying binder levels. Dou et al. [28] studied different fractal models that combined the pore structure and the permeability of tight sandstones. They used mercury intrusion porosimetry to study the correlation between the pore characteristics and the permeability of sandstones. It is clear from literature and theory that the larger the pore radius, the larger the permeability of the material is [29]. Some important characteristics of the pore network such as the critical pore diameter of a porous material can be obtained from the mercury intrusion porosimetry. The critical pore diameter is the smallest diameter of pore that form interconnected voids. It is the minimum diameter after which the porous structure is most interconnected (most gases are evacuated through pores larger than this diameter [30,31,32]). The critical pore diameter affects the permeability of the material. The critical pore diameter has been considered as the point of abrupt variation in the intrusion curves by some authors [33], while some authors suggest the critical pore diameter is obtained from the inflection point. The inflection point is the highest point in the differential intrusion curve. There is therefore a great deal of uncertainty in the method used for the determination of the critical pore diameter. Due to the variation in the material and pore structure, this ambiguity is found to be common when interpreting the mercury intrusion porosimetry data.

## 3. Materials and Methods

The furan sand samples used for this study were prepared using silica sand sourced from a Swedish sand mine called Baskarp and prepared by Sibelco. The aggregate is primarily found to have sub-angular shaped grains [34,35]. Sieve analysis was performed as the first step to identify the grain size distribution. The sieve analysis was performed using a laboratory sieve shaker manufactured by Multiserw-Morek.

**Figure 1:**
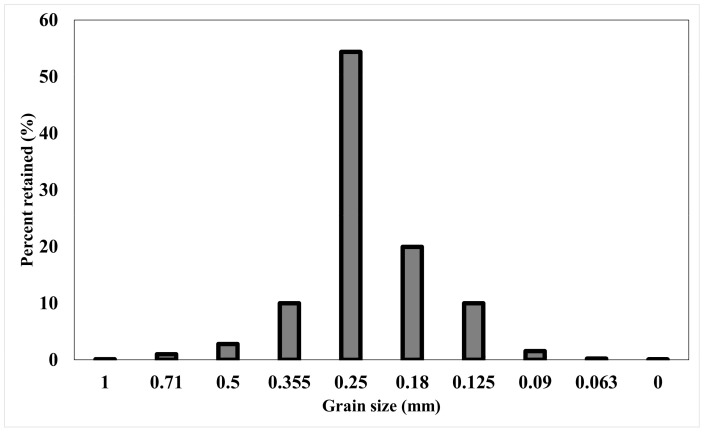
Results of the sieve analysis of the original sand used to prepare samples.

The grain size distribution of the aggregate is shown in Figure 1. The sieved fractions were collected into separate bags mixed according to the proportions mentioned in the Table 1. The appellation of fractions follow the sieve opening number but should be considered as a grain size range up to the next sieve size. For example, 0.125 mm fraction includes grains bigger than 0.125 mm retaining on the sieve with 0.125 mm opening, but smaller than 0.18 mm as it falls through the sieve with 0.18 mm opening.

By modifying the amount of 0.125 mm fraction, the goal was to bring about a difference between the average grain size of the samples. The proportions and percentage of each grain size present in the three prepared batches are shown in Table 1. The amount of 0.125 mm fraction was altered for the three batches to 0, 20, and 40%, respectively. The amounts of other grains were altered accordingly.

**Figure 2:**
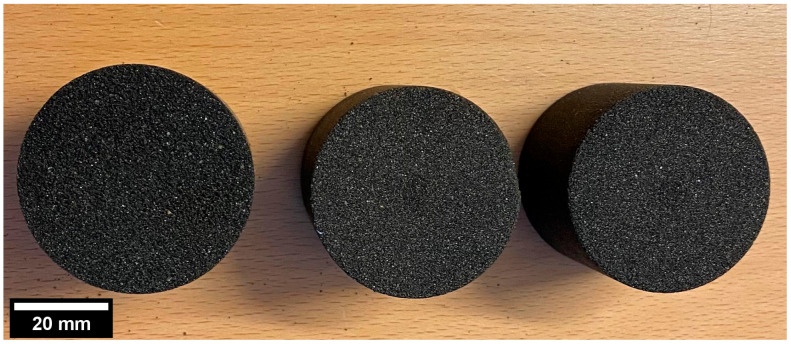
Furan sand samples with varying grain size distributions.

The prepared batches were mixed individually with 2% percent furan resin and a sulphonic acid-based catalyst (40% of the mass of the resin) to aid the polymerization process. The mixture was then compacted using custom made sample holders and rammers to standard cylindrical samples of 50 × 50 mm. The curing time was 24 h for these samples. A rammer with a stopper ensured constant dimensions for the samples. However, due to the manual compaction, there was a very slight difference in the density values. The cylindrical samples (Figure 2.) that were prepared from the mixtures of each batch were named A, B, and C (referring to the nomenclature of the sand batches in Table 1). Table 2 shows the properties of the samples studied. The average grain size for the samples was calculated according to the Swedish standard and is presented in Table 2.

**Figure 3:**
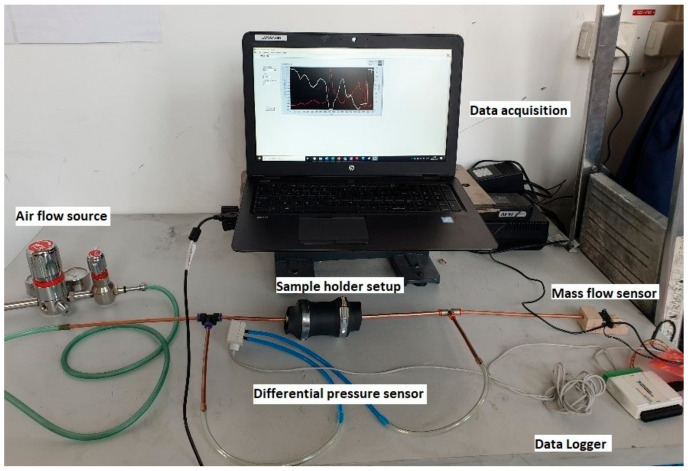
Custom made permeability measurement setup (image reproduced from international journal of cast metals research, published by Taylor and Francis, 2021) [10].

The permeability of these samples was studied using a custom-made measurement system prepared at the School of Engineering of Jonkoping University [11]. The experimental setup is shown in Figure 3.

The setup includes a sample holder to hold the sand core, airflow tubes, differential pressure sensors, airflow meter, and data acquisition system. The sample is wrapped to the holder setup using a heat shrink tube that provides an air-tight enclosure of the sand core. Air from the compressor is passed through flow tubes and regulated using a pressure regulator and flow valve to control the airflow rate. A differential pressure sensor is placed between the input and the output side of the sample. This sensor had a range of 0–2 kPa. The flow rate at the outlet end was measured using a flow sensor with a measuring range of 0–1 m/s of airflow. The volumetric airflow rate was obtained from the velocity measurements. The pressure was altered in the regulator during each measurement so that there is an increase in the flow rate. After each flow increase, there was a change in the differential pressure between the upstream and the downstream ends.

The flow rate, Q, and the pressure difference, Δp, were found to be in the Darcy flow regime. For each sample, a total of 5 measurements were performed to obtain pressure drop and outlet velocity values. The variance and standard deviations were calculated for the samples to avoid any experimental error. After the measurement, the samples were preserved carefully for the porosimetry sample preparation. In foundry technology, the ability to predict the permeability of a foundry core using the pore structure properties will be useful for understanding the flow behavior of cores and molds. Such relationships will need quantification of the pore structure properties. Additionally, permeability in standard units will help calculate the mass and heat transfer in these porous structures.

The methods used for studying the pore characteristics and permeability of materials were introduced earlier. Mercury porosimetry technique is the most widely used liquid intrusion techniques used for pore structure analysis. This technique works based on the non-wetting nature of mercury and the higher contact angle when it comes in contact with a porous surface. In this work, the mercury intrusion porosimetry technique is used for the measurement of the pore structure of furan bonded cores.

**Figure 4:**
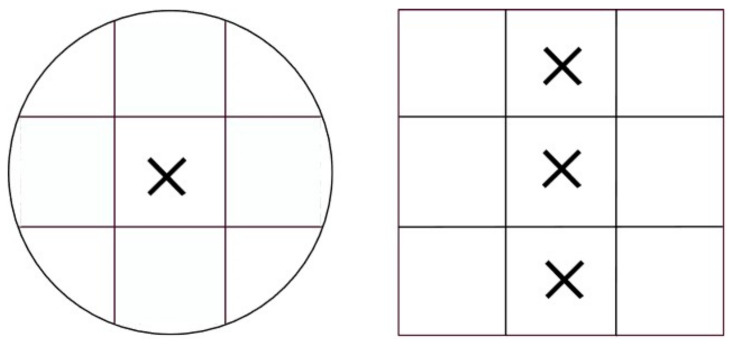
Positions of the samples cut from the cylindrical furan samples for Mercury intrusion porosimetry. ‘x’ corresponds to the porosimetry sample’s center.

The samples preserved after the permeability measurement were then cut to prepare samples for the Mercury intrusion porosimetry study. Mercury intrusion porosimetry (MIP) was performed using the equipment, Micro metrics Autopore Ⅲ 9410 at the research institute, RISE, Sweden. The surface tension and contact angle of mercury were set to 485 mN/m and 130°, respectively. Three positions from each sample were identified and cut from the cylindrical furan sand sample. The positions of the sample are shown in Figure 4.

The sample size was determined based on the dimensions of the specimen holder of the MIP equipment. The dimensions of the porosimetry samples were calculated based on the intrudable pore volume and samples from the cylindrical furan sample of 14 × 14 × 14 mm cut from it. Hence, a total of 3 samples from each cylinder were cut and prepared such that they had accuracy in terms of dimensions and weight. As a requirement for the MIP, the samples were dried in a furnace for 24 h to eradicate the free moisture.

## 4. Results

### 4.1. Permeability

**Figure 5:**
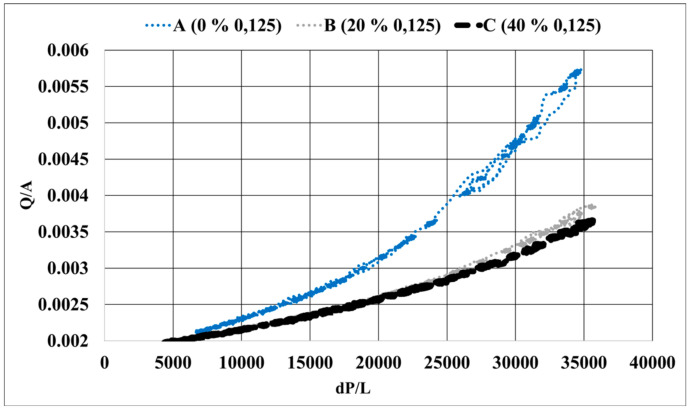
Q/A plotted against dP/L for the three samples measured.

The ratio of volumetric flow rate as a function of the cross-sectional area of the sample to the differential pressure measured as a function of the sand core’s length is shown in Figure 5. The plot shows the super-imposed curves for the three different samples measured. The slope of these curves provides the ratio of permeability to the dynamic viscosity, of the samples, K/µ.

**Figure 6:**
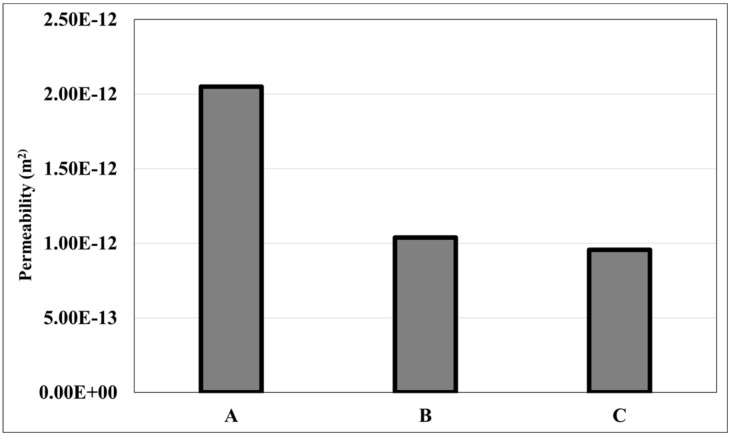
Permeability of the furan samples measured.

The permeability, K, obtained from this relationship is shown in Figure 6. The permeability increased with increasing average grain size and decreasing amount of 0.125 mm fraction. Sample A, which had no 0.125 grains, showed the highest permeability value of 2.05 × 10^−12^ m^2^. Sample B, with 20% 0.125 mm fraction, showed a permeability value of 1.04 × 10^−12^ m^2^, and Sample C, with 40% 0.125 mm, showed permeability values slightly lower than B with 9.56 × 10^−12^ m^2^.

The result shows clearly that an increasing percentage of fine-sized grains (0.125 mm) decreased the permeability. However, the difference between samples B & C, was not significant, implying that the permeability has flattened out after a certain threshold value of increased fineness. The results are in accordance with previous works and theory, where permeability decreased with decreasing average grain size. Data about the pore geometry is critical to make further implications on the results obtained.

### 4.2. Porosimetry

The mercury intrusion porosimetry was performed at RISE IVF, Sweden. A total of 9 specimens were studied. Three pieces were taken from different positions of A, B, and C furan samples, respectively (refer to Figure 5). The direction of compaction was not part of the scope of this study and hence the positions do not necessarily follow the order 1, 2 and 3 based on the direction of compaction. Generally, it could be seen that the studied furan samples have only macropores according to the IUPAC classification. The results showed slight differences in the pore structure properties between the three specimens of the same sample.

**Figure 7:**
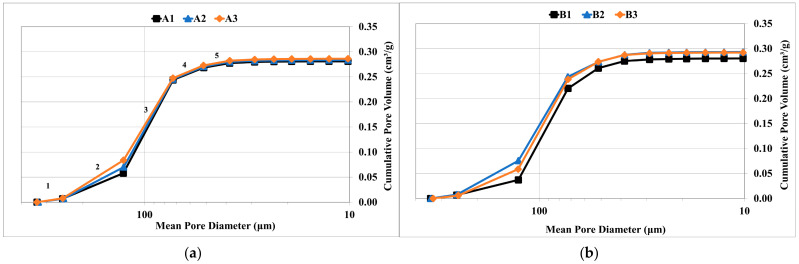

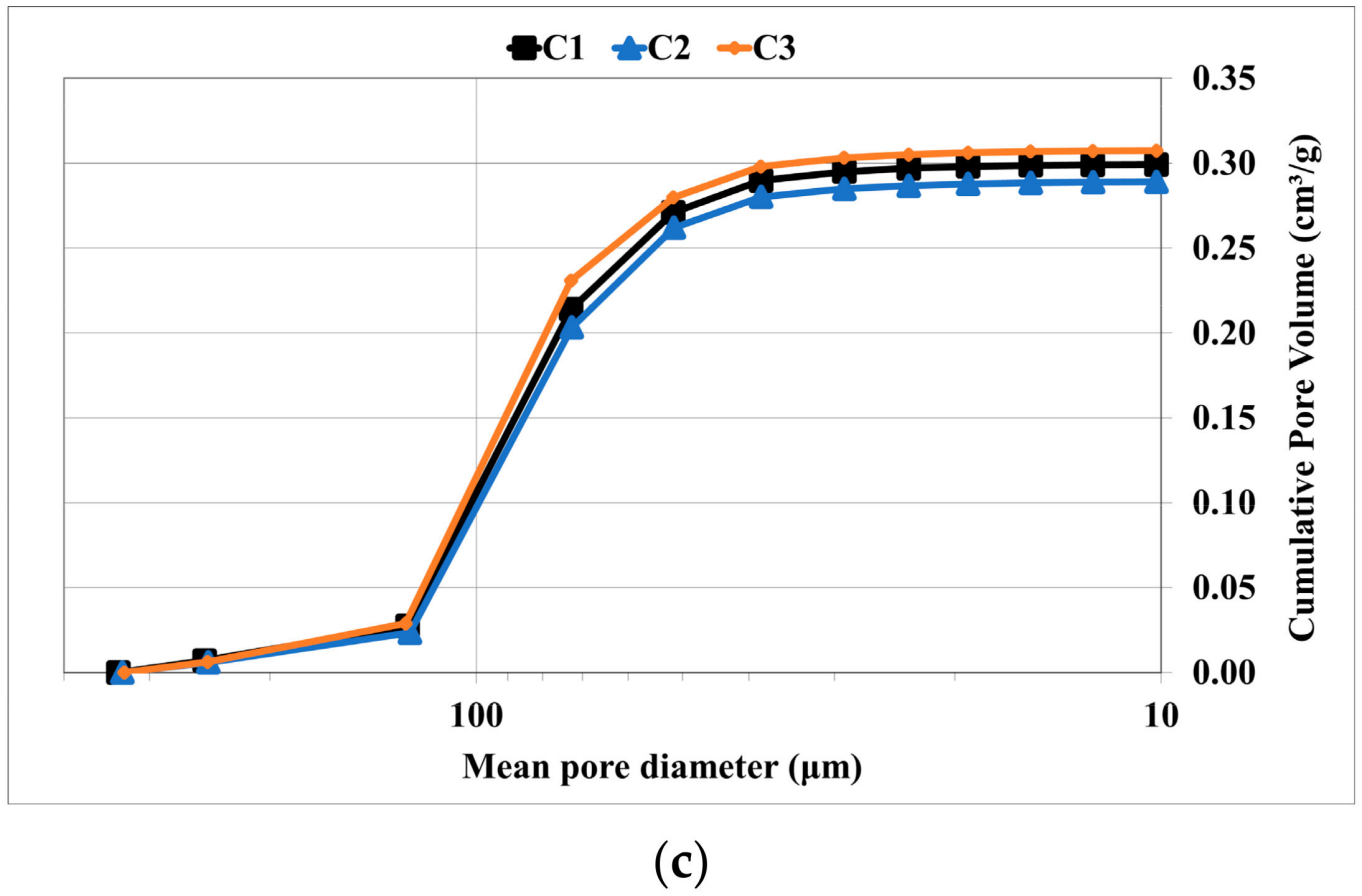
Cumulative volume intrusion curves for three specimens of sample (**a**) A with 0% 0.125 (**b**) B with 20% 0.125. (**c**) C with 40% 0.125.

Figure 7a–c shows the cumulative pore volume against the mean pore diameter for the three specimens of sample A, B and C respectively. The scale is altered such that only the primary parts of the intrusion curve are presented. The pore size ranges could be seen from the 5 major positive slopes in the intrusion curve marked in Figure 7a. The five major slopes and their corresponding pore size ranges are presented in Table 3. For all the 9 specimens studied, slope 3 exhibits the steepest increase in pore volume, which means, the samples have highest number of pores in the size range of 75−130 μm.

The slope 2 with a pore range 130–250 μm is interesting to notice for samples A and B. In A, the slope of A3 is steeper than A1 and A2, whereas A1 has the relatively flattest slope. It is obvious that in the pore range between, 130–250 μm, the A3 has intruded more volume than A1. This implies that the concentration of pores in this range is higher for A3 than A1. Hence the median pore diameter for A1 is slightly lower than A3 (70 and 73 μm respectively). The same trend is seen between B1 and B2, where B1 slope was relatively flatter than B2 and eventually B1 had a lower median diameter than B2 (67 and 71 μm respectively). The data for the pore diameters of individual samples of A, B and C are presented in Table 4.

Since the manually compacted foundry cores show a certain level of anisotropy, this result is unsurprising. The varying pore properties between the same sample show that the direction of compaction affects the pore characteristics in the different parts of the mold. Due to this, the median pore diameter has varied for the specimens of the same cylindrical sample. This effect needs to be considered in foundries because different parts of mold could exhibit different ability to evacuate gases. This behavior could result in a casting that is more prone to gas defects in certain parts, since it is in contact with a mold that possesses different pore structures in different parts (determined by the direction of compaction). The pore characteristics of the nine specimens are presented in Table 4.

**Figure 8:**
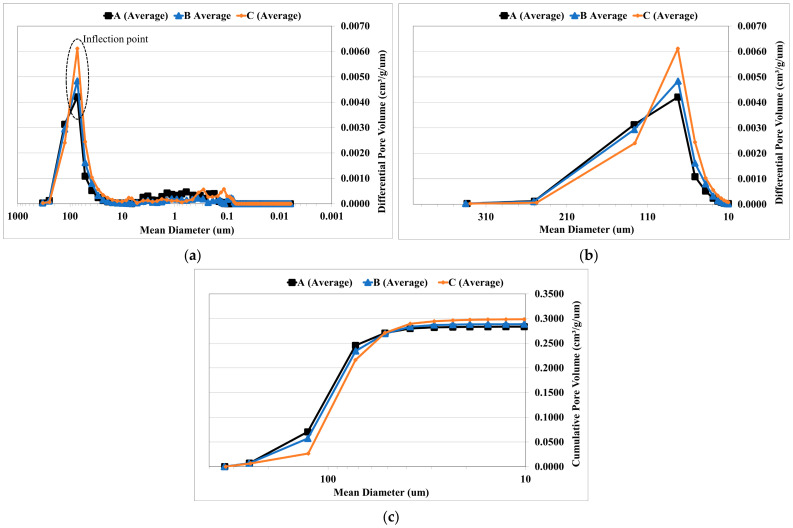
(**a**) Differential volume intrusion curves for three samples A, B and C. (**b**) Differential intrusion curves for the three samples with a linear *x*-axis. (**c**) Cumulative intrusion curves for the three samples A, B and C.

The results of the three samples with varying grain size distributions are discussed with curves prepared from the average of the three individual specimens (Figure 8a–c). If the inflection point is to be considered as the critical pore diameter, there is no variation, in three different sample types A, B and C and their corresponding positions. The inflection point is the highest point in the differential intrusion curve and the value is approximately 76 μm for all the specimens (Figure 8a). Hence it could be concluded from this analysis that the critical pore diameter does not vary much between the samples.

However, the most interesting result from the porosimetry is the difference in the concentration of the pores that is represented by the area under the differential intrusion curve. (Figure 8b). The diversity could be noted also from the steepness of the slope on the cumulative intrusion curve (Figure 8c). The sample A with 0% 0.125 fraction had a higher concentration of the larger pores (250–130 μm), and lower concentration of smaller pores (130–75 μm). Sample B, when compared with A showed a slightly lower number of pores for both the categories. However, sample C, showed a different trend where the larger pores were lower in concentration and the smaller pores are higher in concentration. This corresponds with the median pore diameter data presented in Table 5 (Samples A, B and C).

In summary, sample C has a greater number of smaller pores and has a higher total porosity. It also has the lowest median pore diameter. This is not the case with sample A and B, where the pore diameter was higher while these samples exhibited slightly lower porosities. The pore diameter is vital and influences the permeability because the pore diameter affects the velocity of the fluid passing through the sample and the differential pressure between the inlet and the outlet side of the sample.

### 4.3. Relationship between Permeability and Pore Characteristics

**Figure 9:**
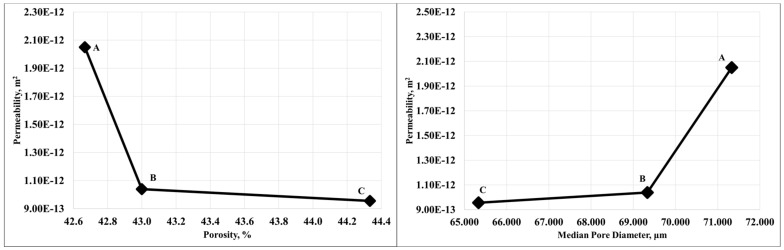
(**a**). Permeability as a function of the median pore diameter. (**b**) Permeability as a function of the total porosity.

The results of the porosimetry when correlated with the permeability results showed the expected relationship where the permeability decreased with decreasing pore diameter. Figure 9a shows the results of permeability as a function of median pore diameter. Contrarily, permeability was seen decreasing for sample with increasing porosity. Permeability as a function of the total porosity is plotted in Figure 9b.

The pore volume and total porosity is on an increasing trend for A, B and C with respect to the average grain size. At first, it seems counter-intuitive that the samples with higher fine fraction content show higher total porosity. At the same time, the bulk density of sample B and C are slightly lower, than for sample A (refer Table 5). Granulous material with smaller grain size requires greater compaction force to achieve the same bulk density and strength. The fact that the samples in this study has been prepared with manual compaction explains the lower bulk density and hereby higher porosity for the samples with more fines in it. However, the median pore diameter showed that the samples with increased fines have reduced permeability, owing to the presence of a greater number of smaller pores and increased pressure build up. Table 6 shows the permeability data along with the pore characteristics.

This result shows that even though a granular system with a smaller average grain size should have lower porosity, in practice/foundry technology, when the compaction force is constant in a molding line, the bulk density of molding mixtures with more fines will be lower, and so the mold/core will have higher porosity but a lower permeability because of a smaller average pore diameter.

## 5. Conclusions

In this work, chemically bonded sand samples prepared using furan binder system were studied. The grain size distribution of the samples was varied by altering the percentage of 0.125 mm grains. Permeability of furan samples with varying 0.125 fraction was measured using a custom-made setup to find the effect of grain size distribution on the permeability in standard units of m^2^.

The results of the permeability measurements showed that with 20% increase of 0.125 fraction, the permeability decreased significantly, while a further 20% increase in the 0.125 fraction did not significantly decrease the permeability of the sample. From this result, it is clear that permeability has a threshold limit in terms of amount of finer grains added to the sample. The permeability measurements were followed by mercury intrusion porosimetry measurements for three samples on three positions.

Although pores in a molding mixture vary in size from 350 μm to 100 nm, most pores are in the range of 130–50 μm. Alteration in the grain size distribution does not affect the critical pore diameter which is the smallest diameter of pore that form interconnected voids in a molding mixture. Compaction direction results in anisotropy of pore characteristics. The slight variation in terms of porosity and median pore diameter for the same sample shows that during the casting process, the mold could outgas differently at different parts. The total porosity could be higher for a mold with a finer fraction, but it could possess pores with smaller diameter leading to a lower permeability. This study therefore has shown that the permeability is more dependent on the pore diameter and that porosity has a secondary influence on permeability. In foundry practice, in a molding line where the compaction force is constant, the bulk density of a molding mixture with higher fines will be lower, leading to a higher porosity. While such molding mixtures will have smaller pore diameters and lower permeability values and have difficulty in evacuating the volatile compounds evolving during the casting process.

## Figures and Tables

**Table 1:** Proportion of different fractions mixed to obtain the three batches of measurement.

Sieve Opening, mm	Amount of Fraction, %			
	Basic Sand	A	B	C
1.000	0.104	0.12	0.09	0.07
0.710	0.988	1.10	0.88	0.66
0.500	2.808	3.12	2.50	1.87
0.355	9.980	11.09	8.87	6.65
0.250	54.350	60.39	48.31	36.23
0.180	19.932	22.15	17.72	13.29
**0.125**	10.000	**0.00**	**20.00**	**40.00**
0.090	1.540	1.71	1.37	1.03
0.063	0.222	0.25	0.20	0.15
0 (PAN)	0.077	0.09	0.07	0.05

**Table 2:** Properties of the studied furan samples.

Sample	Amount of 0.125 mm Fraction (%)	Density (g/cm^3^)	Average Grain Size (mm)
A	0	1.454	0.33
B	20	1.459	0.28
C	40	1.478	0.24

**Table 3:** Pore size range of the 9 molding material specimens.

Slope	Pore Size Range (µm)
1	250–340
2	130–250
**3**	**75–130**
4	52–75
5	40–52

**Table 4:** Results of the mercury porosimetry measurements of the furan samples with varying grain size distributions for all the nine specimens.

Property	Samples		
	A1	A2	A3
Pore volume (cm^3^/g)	0.28	0.29	0.29
Median pore diameter (µm)	70.00	71.00	73.00
Porosity (%)	42.00	43.00	43.00
Bulk density (g/cm^3^)	1.49	1.50	1.50
True density (g/cm^3^)	2.57	2.61	2.63
	**B1**	**B2**	**B3**
Pore volume (cm^3^/g)	0.28	0.29	0.29
Median pore diameter (µm)	67.00	71.00	70.00
Porosity (%)	42.00	44.00	43.00
Bulk density (g/cm^3^)	1.51	1.48	1.49
True density (g/cm^3^)	2.62	2.63	2.64
	**C1**	**C2**	**C3**
Pore volume (cm^3^/g)	0.30	0.29	0.31
Median pore diameter (µm)	65.00	65.00	66.00
Porosity (%)	44.00	44.00	45.00
Bulk density (g/cm^3^)	1.48	1.51	1.47
True density (g/cm^3^)	2.65	2.70	2.70

**Table 5:** Results of the mercury porosimetry measurements of the Furan samples with varying grain size distributions.

Property	Sample		
	A	B	C
Pore volume (cm^3^/g)	0.287	0.287	0.300
Median pore diameter (µm)	71.33	69.33	65.33
porosity (%)	42.66	43.00	44.33
Bulk density (g/cm^3^)	1.495	1.494	1.488
True density (g/cm^3^)	2.602	2.631	2.684
Percent intrusion at d_crit_ (%)	86.25	80.87	72.07
d_crit_ (µm)	72.66	72.49	72.64

**Table 6:** Permeability of samples A, B and C presented along with the average porosimetry data for the three samples.

Property	Sample		
	A	B	C
Median pore diameter (µm)	71.33	69.33	65.33
porosity (%)	42.66	43.00	44.33
Permeability, m^2^	2.05 × 10^−12^	1.04 × 10^−12^	9.56 × 10^−13^
Average grain size (mm)	0.33	0.28	0.24

## Data Availability

Data sharing not applicable.

## Nomenclature

**Small letters**	
*dp*	pressure gradient, Pa
*r*	pore radius, μm
**Capital letters**	
*A*	Cross-sectional area of the porous material, m^2^
*L*	Path length of the porous material, m
*Q*	Volumetric flow rate, m^3^ s^−1^
*K*	Darcian permeability, m^2^
*P*	Pressure, MPa
**Greek symbols**	
*ρ*	density of the fluid, g cm^−3^
*μ*	dynamic viscosity, kg m^−1^s^−1^
ε	porosity, %
ϒ	surface tension (interfacial energy) of mercury, N m^−1^
⍬	Contact angle of mercury with the material, deg.

## References

[1] Campbell J. (2018). Molding and Casting Processes. Cast Iron Sci. Technol..

[2] Svidró J.T., Diószegi A., Tóth J. (2014). The novel application of Fourier thermal analysis in foundry technologies: Examination of degradation characteristics in resin-bound molding materials. J. Therm. Anal. Calorim..

[3] Campbell J. (2011). Molds and cores. Complete Casting Handbook.

[4] Des Plaines I.I.I. (2001). AFS Mold & Core Test Handbook.

[5] Darcy H.P.G. (1856). Les Fontaines Publiques de la Ville de Dijon.

[6] Winardi L., Littleton H., Bates C.E. (2005). New Technique for Measuring Permeability of Cores Made from Various Sands, Binders, Additives and Coatings. Trans. Am. Foundry Soc..

[7] Wisniewski P., Sitek R., Towarek A., Choinska E., Moszczynska D., Mizera J. (2020). Molding binder influence on the porosity and gas permeability of ceramic casting molds. Materials.

[8] Kumar S., Karunakar D.B. (2019). Enhancing the Permeability and Properties of Ceramic Shell in Investment Casting Process Using ABS Powder and Needle Coke. Int. J. Met..

[9] Chate G.R., Patel G.C.M., Kulkarni R.M., Vernekar P., Deshpande A.S., Parappagoudar M.B. (2018). Study of the Effect of Nano-silica Particles on Resin-Bonded Molding Sand Properties and Quality of Casting. Silicon.

[10] Sundaram D., Svidró J.T., Diószegi A., Svidró J. (2021). Measurement of Darcian Permeability of foundry sand mixtures. Int. J. Cast Met. Res..

[11] Mádi L., Varga L. (2018). The effect of gas permeability on the pressure of artificial resin-bonded core gases. IOP Conf. Ser. Mater. Sci. Eng..

[12] Mádi L., Budavári I., Varga L. (2020). The effect of different grain sizes and heat input on the gas pressure inside artificial resin-bonded sand cores. IOP Conf. Ser. Mater. Sci. Eng..

[13] Sahoo P.K., Pattnaik S., Sutar M.K. (2020). Investigation of the Foundry Properties of the Locally Available Sands for Metal Casting. Silicon.

[14] Kashima J. (1968). Relation between permeability and grain size of molding sands. J. Jpn. Foundrymen’s Soc..

[15] Nield D.A., Bejan A. (2012). Convection in porous media: Fourth edition. Convection in Porous Media.

[16] Mitra S., EL Mansori M., Rodríguez de Castro A., Costin M. (2020). Study of the evolution of transport properties induced by additive processing sand mold using X-ray computed tomography. J. Mater. Process. Technol..

[17] Marshall T.J. (1958). A relationship between permeability and size distribution of pores. J. Soil Sci..

[18] Ettemeyer F., Lechner P., Hofmann T., Andrä H., Schneider M., Grund D., Volk W., Günther D. (2020). Digital sand core physics: Predicting physical properties of sand cores by simulations on digital microstructures. Int. J. Solids Struct..

[19] Vasková I., Varga L., Prass I., Dargai V., Conev M., Hrubovčáková M., Bartošová M., Buľko B., Demeter P. (2020). Examination of behavior from selected foundry sands with alkali silicate-based inorganic binders. Metals.

[20] Van Keulen J. (1973). Density of porous solid, (RILEM). Mater. Struct..

[21] Espinal L. (2012). Porosity and Its Measurement. Characterization of Materials.

[22] Washburn E.W. (1921). The dynamics of capillary flow. Phys. Rev..

[23] Sing K., Everett D., Haul R., Moscou L., Peirotti R., Rouquerol J., Siemieniewska T. (1985). IUPAC commission on colloid and surface chemistry including catalysis. Pure Appl. Chem..

[24] Fang X., Cai Y., Liu D., Zhou Y. (2018). A Mercury Intrusion Porosimetry Method for Methane Diffusivity and Permeability Evaluation in Coals: A Comparative Analysis. Appl. Sci..

[25] Dong H., Zhang H., Zuo Y., Gao P., Ye G. (2018). Relationship between the Size of the Samples and the Interpretation of the Mercury Intrusion Results of an Artificial Sandstone. Materials.

[26] Pittman E.D. (1992). Relationship of porosity and permeability to various parameters derived from mercury injection-capillary pressure curves for sandstone. Am. Assoc. Pet. Geol. Bull..

[27] Martinez D., Bate C., Manogharan G. (2020). Towards Functionally Graded Sand Molds for Metal Casting: Engineering Thermo-mechanical Properties Using 3D Sand Printing. JOM.

[28] Dou W., Liu L., Jia L., Xu Z., Wang M., Du C. (2021). Pore structure, fractal characteristics and permeability prediction of tight sandstones: A case study from Yanchang Formation, Ordos Basin, China. Mar. Pet. Geol..

[29] Tian R. (2017). A Theoretical Analysis of Pore Size Distribution Effects on Shale Apparent Permeability. Geofluids.

[30] Zhu J., Zhang R., Zhang Y., He F. (2019). The fractal characteristics of pore size distribution in cement-based materials and its effect on gas permeability. Sci. Rep..

[31] Katz A.J., Thompson A.H. (1986). Quantitative prediction of permeability in porous rock. Phys. Rev. B Condens. Matter.

[32] Nishiyama N., Yokoyama T. (2017). Permeability of porous media: Role of the critical pore size. J. Geophys. Res. Solid Earth.

[33] Guimarães A.T.D.C., De Vera G., Rodrigues F.T., Antón C., Climent M.A. (2015). Comparison between dcrit considering the abrupt variation and inflexion in the concrete mercury intrusion porosimetry curve. Exp. Tech..

[34] Svidró J.T., Diószegi A., Svidró J., Ferenczi T. (2017). Thermophysical aspects of reclaimed molding sand addition to the epoxy-SO2 coremaking system studied by Fourier thermal analysis. J. Therm. Anal. Calorim..

[35] Svidró J., Diószegi A., Svidró J.T. (2020). The origin of thermal expansion differences in various size fractions of silica sand. Int. J. Cast Met. Res..

